# Dietary *Lepidium meyenii* Supplementation Is Associated with Hepatic Transcriptomic and Targeted DNA Methylation Changes in Pigs

**DOI:** 10.3390/genes17091128

**Published:** 2026-09-16

**Authors:** Weronika Loba-Pasternak, Anita Zaworska-Zakrzewska, Joanna Nowacka-Woszuk

**Affiliations:** 1 Department of Genetics and Animal Breeding, Poznan University of Life Sciences, Wolynska 33, 60-637 Poznan, Poland; weronika.loba@up.poznan.pl; 2 Department of Animal Nutrition, Poznan University of Life Sciences, Wolynska 33, 60-637 Poznan, Poland; anita.zaworska-zakrzewska@up.poznan.pl

**Keywords:** Peruvian maca, pig, liver, RNA sequencing, gene expression, DNA methylation, Western blot, hepatic metabolism

## Abstract

**Background**: *Lepidium meyenii* Walp. (maca) is a plant-derived feed supplement rich in bioactive compounds such as macamides, macaenes, glucosinolates, and polyphenols. In this study, it was selected as a potential natural functional feed additive based on its bioactive phytochemical composition, reported metabolic properties, and previously observed effects on porcine cells. However, its molecular effects in livestock remain poorly characterised. This study investigated hepatic molecular responses to dietary maca supplementation in pigs using transcriptomic analysis followed by DNA methylation, promoter polymorphism, and protein analyses to explore the mechanisms underlying the observed transcriptional changes. **Methods**: Female pigs were assigned to two groups fed a diet supplemented with 1% maca powder (E) or a control diet (C). Liver samples were analysed using RNA sequencing and qPCR validation of selected genes. DNA methylation, promoter variants, and TUBA4A protein abundance were assessed using pyrosequencing, Sanger sequencing, and Western blotting, respectively. **Results**: RNA-seq identified 104 differentially expressed genes (DEGs) after DESeq2 analysis with intensity difference filtering. qPCR validated the differential expression of *INHBE*, *PPP1R3C*, *PPP1R3B*, *TUBA4A*, and *GCK*. Gene Ontology enrichment analysis indicated the over-representation of terms related to extracellular matrix organisation, collagen-containing structures, response to transforming growth factor beta, and glycogen biosynthetic processes. Methylation analysis revealed no significant methylation changes in *GCK*, *INHBE*, *PPP1R3C*, or *TUBA4A* after FDR correction, whereas *PPP1R3B* showed significant differences at seven CpG sites. Promoter polymorphisms were identified in *PPP1R3C* and *TUBA4A*, but genotype frequencies did not differ between groups. Additionally, TUBA4A protein abundance was not altered. **Conclusions**: Dietary maca supplementation was associated with moderate hepatic molecular changes, including alterations in genes and pathways related to glycogen metabolism and extracellular matrix organisation. Maca may represent a promising natural feed supplement for pigs, although further studies are needed to confirm its efficacy and practical applicability.

## 1. Introduction

The growing interest in sustainable livestock production has intensified the search for natural feed additives to improve animal performance, health, and product quality. Among plant-derived supplements, *Lepidium meyenii* Walp. (maca), which is a species native to the Peruvian Andes, has gained attention due to its rich phytochemical composition and functional properties. Traditionally consumed as a food and medicinal plant, maca is characterised by the presence of biologically active compounds such as macamides, macaenes, glucosinolates, alkaloids, and polyphenols [1,2,3,4]. These compounds are associated with antioxidant, metabolic, and endocrine-modulating effects [1,2]. The biological activity of maca is likely related to the combined action of several classes of secondary metabolites. Macamides are characteristic lipophilic N-benzylamides of long-chain fatty acids and are considered among the most distinctive bioactive compounds of dried maca. Macaenes are polyunsaturated fatty acid derivatives that occur together with macamides and have been associated with the antioxidant activity of maca, although their specific molecular targets remain less well characterised [5]. Glucosinolates, which are characteristic secondary metabolites of Brassicaceae plants, can be hydrolysed to biologically active products such as isothiocyanates. These metabolites may modulate cellular antioxidant and detoxification responses and influence inflammatory and metabolic signalling [6]. Maca also contains polyphenolic compounds, which may contribute to its antioxidant properties through free radical scavenging and protection against oxidative stress [7]. Therefore, rather than acting through a single constituent, maca supplementation may affect cellular metabolism through the combined activity of several phytochemical classes with antioxidant and regulatory properties. Experimental studies suggest that maca supplementation may influence physiological processes relevant to animal production, including reproductive function and metabolic regulation [6,8,9]. The antioxidant capacity of *L. meyenii* is of particular interest in intensive swine production, in which oxidative stress can impair physiological homeostasis and reduce performance.

Previous studies have demonstrated that maca’s secondary metabolites, especially macaenes and macamides, may scavenge free radicals and increase the expression of endogenous antioxidant enzymes, thus potentially mitigating the harmful effects of reactive oxygen species on muscle tissue stability [3,4].

Despite the growing interest in maca as a functional feed additive, knowledge of its molecular effects in livestock remains limited, particularly at the transcriptomic, epigenetic, and proteomic levels. In pigs, the liver is a central organ linking nutrient utilisation, glucose homeostasis, lipid metabolism, and systemic endocrine signalling. Dietary bioactive compounds such as macamides and macaenes may therefore produce hepatic molecular effects even when classical performance or carcass traits show only limited changes [10]. Transcriptomic analysis provides a wide overview of these responses, but changes in mRNA abundance do not always translate into altered protein levels and may be influenced by epigenetic mechanisms. For this reason, combining RNA-seq with qPCR validation, targeted DNA methylation analysis, genetic variant searching, and protein level measurement may provide a more reliable interpretation of the biological response to dietary supplementation.

Our interest in *L. meyenii* as a potential feed additive for pigs was motivated by its reported metabolic and antioxidant properties and our previous in vitro findings showing that maca extract modulated proliferation and adipogenesis in porcine cells [11]. These observations suggested that maca may influence metabolic processes relevant to growing pigs, while its molecular effects following dietary supplementation in vivo remain largely unexplored. This highlights the need for comprehensive in vivo studies to elucidate the systemic molecular responses to maca supplementation in livestock species, particularly pigs. Special attention should be given to genes representing different aspects of hepatic physiology. The selected panel includes genes involved in glycogen metabolism (*PPP1R3B*, *PPP1R3C*, and *GCK*), endocrine and metabolic signalling (*INHBE* and *IGFALS*), lipid metabolism (*SREBF1*), extracellular matrix organisation (*DCN*), epithelial barrier function (*CLDN4*), and cytoskeletal organisation (*TUBA4A*).

Collectively, these genes provide a broad overview of the molecular pathways potentially influenced by dietary maca supplementation. Among them, *PPP1R3B* encodes a glycogen-targeting regulatory subunit of protein phosphatase 1 (PP1) and plays an important role in hepatic glycogen storage and whole-body glucose and energy homeostasis [12]. *INHBE* encodes activin E, which is a liver-derived member of the TGF-beta superfamily that has recently been implicated in liver–adipose tissue communication and metabolic regulation [13,14]. Thus, changes in these genes may indicate that maca supplementation affects hepatic metabolic signalling.

The aim of the present study was to investigate whether dietary supplementation with *L. meyenii*, which was selected based on its reported bioactive and metabolic properties and our previous findings in porcine cells, affects gene expression, DNA methylation, and protein abundance in growing pigs. This study combined RNA-seq with qPCR validation, pyrosequencing analysis, and Western blotting to provide comprehensive insight into the molecular responses associated with maca supplementation.

## 2. Materials and Methods

### 2.1. Ethics Statement

Animal husbandry and slaughter were carried out under routine commercial farming conditions and in accordance with applicable animal welfare regulations. No invasive procedures or experimental interventions requiring specific ethical approval were performed on the animals, and the standard commercial slaughter procedure was not modified for the purposes of this study. Biological material was collected during routine slaughter in accordance with standard commercial procedures. Therefore, in line with applicable national regulations, separate approval from the local ethical committee for animal experimentation was not required. Nevertheless, relevant animal welfare guidelines were followed throughout this study. During the rearing period, the pigs remained under regular veterinary supervision and had ad libitum access to feed and water.

### 2.2. Plant Material

The powdered root of Peruvian maca (*L. meyenii*), originating from Peru, was obtained from a local distributor (BIO PLANET S.A., Leszno, Poland). Prior to this experiment, the material was stored in a cool, dry, and dark environment in accordance with the manufacturer’s storage recommendations provided on the product label. The chemical composition and bioactive compound profile of the Peruvian maca powder used, were characterised in detail in our previous study [10]. The analysed components included antioxidant activity, total phenolic and flavonoid contents, individual phenolic compounds, macamides, fatty acid derivatives, and sterols.

### 2.3. Animal Experiment

This study was conducted on a commercial pig farm in the Wielkopolska region of Poland. A total of 40 female piglets with an initial body weight of about 29 kg were used. The animals were housed in group pens and randomly divided into two groups (n = 20 each): an experimental group (E) receiving a diet supplemented with 1% Peruvian maca and a control group (C) without supplementation. Each pig was identified with an individual ear tag. It should be acknowledged that the present study was conducted under commercial farm conditions, with 20 pigs assigned to each dietary treatment and housed in a single group pen per treatment. Therefore, the results should be interpreted with due caution, as the lack of independent pen replicates limits the extent to which the observed effects can be attributed specifically to the dietary treatment. Nevertheless, conducting this study under practical commercial production conditions represents an important strength, as it provides valuable insights into the potential applicability of *L. meyenii* supplementation under standard farming conditions.

This experiment lasted 108 days and included two feeding periods: Phases I (48 days) and II (60 days). The C group was fed a standard diet, while in the E group, 1% of wheat bran (10 kg per tonne of feed) was replaced with maca root powder [10]. Diets were prepared according to recommended nutritional standards for pigs [15]. Animals were checked daily, and their health and welfare were monitored throughout this experiment. The inclusion level of maca was selected based on the limited evidence available from studies conducted in other animal species and corresponded to the upper end of the dietary range previously investigated (0.09–1%). This level was considered appropriate for the initial evaluation of the potential biological effects of *L. meyenii* supplementation in pigs. Wheat bran was selected as the ingredient to be replaced because its proximate chemical composition was comparable to that of maca, allowing for a straightforward 1:1 replacement without substantially altering the basic nutritional value of the diet. This approach enabled the potential effects of maca to be evaluated while minimising the influence of differences in the nutritional composition of the experimental diets. The nutritional value and proximate composition of the maca and feed mixtures used in this experiment were reported in detail in our previous study, as well as detailed information regarding the housing and environmental conditions, including pen characteristics, bedding, temperature, ventilation, and lighting conditions [10].

### 2.4. Sample Collection

When the feeding experiment was finished (at which point the animals’ body weight was around 124 kg), all pigs were transported to an approved slaughterhouse. Prior to slaughter, the animals underwent a standardised fasting period maintained for 12 h, with unrestricted access to water. The time of the final feeding, transport duration, and the interval between arrival at the slaughterhouse and slaughter were recorded. Animals were stunned electrically according to standard procedures and then slaughtered using exsanguination. Liver tissue was collected from all animals (n = 20 per group) shortly after slaughter. Samples were excised from the right lobe, immediately frozen on dry ice, and subsequently stored at −80 °C until molecular analyses.

The number of animals included in individual molecular analyses differed depending on sample availability and quality. RNA-seq was performed on liver samples from 10 animals per group due to the high cost of sequencing. These animals were selected based on their higher RIN (RNA Integrity Number) value. For qPCR analyses, the sample size was increased to 16 animals per group. The same 16 animals per group were subsequently used for Western blot analysis and DNA methylation to maintain consistency between mRNA and protein and methylation level analyses. DNA isolation for Sanger sequencing was successful for all available samples; thus, 20 animals per group were analysed.

### 2.5. RNA Extraction, Library Preparation, and Sequencing

Total RNA was extracted from liver tissue using the RNeasy Mini Kit (QIAGEN, Hilden, Germany). The analysis included samples from 10 animals in both the E and C groups. Each tissue sample was homogenised in RTL buffer using a TissueLyser LT (QIAGEN, Hilden, Germany), according to the manufacturer’s protocol.

RNA purity was checked using a NanoDrop 2000 spectrophotometer (Thermo Fisher Scientific, Waltham, MA, USA), while RNA concentration was measured with the Qubit RNA BR Assay Kit (Thermo Fisher Scientific, Waltham, MA, USA) using a Qubit 2.0 fluorometer (Invitrogen, Carlsbad, CA, USA). For RNA-seq analysis, the RIN was evaluated with the RNA 6000 Nano Kit on an Agilent 2100 Bioanalyzer (Agilent Technologies, Santa Clara, CA, USA). Only samples with RIN values above 8 and a 260/280 ratio higher than 2.0 were used for further analysis. For library preparation, 100 ng of total RNA from each sample was used with the TruSeq Stranded mRNA Kit (Illumina, San Diego, CA, USA). The samples were then sequenced by a commercial facility using the Illumina NovaSeq 6000 system (Illumina, San Diego, CA, USA), generating paired-end reads of 2 × 100 bp.

### 2.6. RNA-Seq Analysis

#### 2.6.1. Initial Bioinformatic Analysis

Paired-end FastQ files were first checked for quality using FastQC (version 0.12.0) [16]. Adapter sequences and low-quality bases were removed with Trim Galore (version 0.6.5) using default settings [17]. The cleaned reads were then mapped to the Sus scrofa 11.1 reference genome using Hisat2 (version 2.2.0), with gene annotation based on Ensembl release 90 [18]. Gene expression levels were calculated in SeqMonk (version 1.48.1 (Babraham Bioinformatics—SeqMonk Mapped Sequence Analysis Tool)) [19], which provided both raw read counts and normalised values expressed as log_2_ reads per million (RPM). Following DESeq2 analysis, an additional Intensity Difference filter implemented in SeqMonk (version 1.48.1) was applied using normalised log_2_-transformed read counts, with a *p*-value < 0.05 used as the significance threshold. This filter identifies genes showing expression differences between groups that exceed the general distribution of intensity differences observed across the dataset. The final candidate gene list was generated by retaining genes identified by both the DESeq2 and SeqMonk Intensity Difference analyses. Differential expression analysis was performed in DESeq2 using dietary treatment as the explanatory factor, with the E and C groups used as the two levels of the factor. No additional covariates were included in the model.

#### 2.6.2. Functional Annotation and Gene Ontology Enrichment Analysis

Gene Ontology (GO) enrichment analysis was performed in the R statistical environment (R version 4.3.1) using the clusterProfiler package (version 4.10.1) together with the org.Ss.eg.db annotation database (version 3.18.0) for *Sus scrofa*. Gene symbols were converted to Entrez Gene identifiers in clusterProfiler. Genes that could not be mapped to Entrez Gene IDs were excluded from further analysis. The default background implemented by enrichGO was used, comprising genes with available GO annotations in the org.Ss.eg.db database. The analysis was performed using a nominal *p*-value cutoff of 0.05 with adjustments made for multiple testing using the Benjamini–Hochberg method.

An over-representation analysis of GO terms was performed using the enrich GO function separately for the three GO ontologies: Biological Process, Molecular Function, and Cellular Component.

### 2.7. qPCR Validation

The qPCR method was used to confirm the RNA-seq results. Nine genes that showed statistical significance in RNA-seq were tested: *CLDN4*, *IGFALS*, *INHBE*, *DCN*, *SREBF1*, *PPP1R3C*, *PPP1R3B*, *TUBA4A*, and *GCK*. *H3F3A* and *PPIA* were used as reference genes. A total of 16 animals from both the E and C groups were used for analysis. A NanoDrop ND-2000 spectrophotometer (Thermo Fisher Scientific, Waltham, MA, USA) was used to assess the concentration and purity of the isolated RNA. Subsequently, 1 μg of total RNA was reverse-transcribed into first-strand cDNA utilising the Transcriptor First Strand cDNA Synthesis Kit (Roche, Indianapolis, IN, USA) in a 20 μL reaction volume. The cDNA was then adjusted with nuclease-free water to achieve a final concentration of roughly 700 ng/µL. Primers were designed using Primer3Plus software (version 3.3.0) (Supplementary Table S1). Quantitative PCR was performed using a LightCycler 480 II system (Roche, Indianapolis, IN, USA) with the LightCycler 480 SYBR Green I Master Kit. Each reaction was carried out in a final volume of 10 µL over 45 cycles. The cycling conditions included initial denaturation at 95 °C for 10 min, followed by denaturation at 95 °C for 10 s, annealing at 60 °C for 10 s, and extension at 72 °C for 10 s. Relative mRNA expression levels were calculated using the standard curve method based on a series of ten-fold cDNA dilutions with known concentration [20]. The results were normalised to the average expression of the reference genes. The amplification efficiencies of the studied genes were determined from standard curves and varied between 81.3% for *H3F3A* and 96.8% for *GASK1*. All reactions were performed in duplicate, and melt curve analysis confirmed a single specific amplification product for each qPCR assay.

### 2.8. Pyrosequencing in Liver Tissue

The analysis aimed at detecting differences in DNA methylation levels was conducted for five genes that exhibited significant variation in the qPCR results. CpG sites were selected based on data obtained from the NCBI Gene database and CpGPlot software (EMBOSS version 6.6.0; http://www.ebi.ac.uk/Tools/seqstats/emboss_cpgplot (accessed on 1 September 2025)). Several CpG sites were identified within the promoters of the *INHBE*, *GCK*, *TUBA4A*, *PPP1R3C*, and *PPP1R3B* genes. The primers used for pyrosequencing (Supplementary Table S2) were designed with PyroMark Assay Design 2.0 software (QIAGEN, Hilden, Germany) to amplify bisulphite-converted target regions. Genomic DNA was extracted from liver tissue collected from 16 E pigs and 16 C pigs using the Genomic Mini Kit (A&A Biotechnology, Gdansk, Poland). DNA concentration and purity were evaluated using a NanoDrop ND-2000 spectrophotometer (Thermo Fisher Scientific, Waltham, MA, USA) based on absorbance ratios at 260/280 nm.

For pyrosequencing preparation, 500 ng of DNA was bisulphite-converted using the EZ DNA Methylation-Gold Kit (Zymo Research, Irvine, CA, USA). Unmethylated control DNA was generated using REPLI-g Mini Kits (QIAGEN, Hilden, Germany) according to the manufacturer’s instructions, while fully methylated controls were prepared by incubating 500 ng of DNA with CpG methyltransferase (M.SssI, Thermo Fisher Scientific, Waltham, MA, USA) at 37 °C for three hours, following the manufacturer’s protocol. PCR amplification was carried out using the PyroMark PCR Kit (QIAGEN, Hilden, Germany) under the following conditions: initial denaturation at 95 °C for 15 min; 44 cycles consisting of denaturation at 94 °C for 30 s, annealing at 56 °C for 30 s, and extension at 72 °C for 30 s; and a final extension step at 72 °C for 10 min. Each run included negative controls without a DNA template. PCR efficiency was verified using electrophoresis on a 1.5% agarose gel. The resulting amplicons were subjected to pyrosequencing using the PyroMark Q48 Autoprep system (QIAGEN, Hilden, Germany) with PyroMark Q48 Advanced CpG Reagents (QIAGEN, Hilden, Germany), in accordance with the manufacturer’s instructions. Methylation percentages at individual CpG sites were calculated using PyroMark Q48 Autoprep software version 2.4.4 (QIAGEN, Hilden, Germany).

### 2.9. Sanger Sequencing

DNA was isolated from liver tissue samples collected from 40 animals, including 20 samples from both the E and C groups, using the Genomic Mini Kit (A&A Biotechnology, Gdansk, Poland). Based on the Sscrofa11.1 pig genome assembly, primers targeting the 5′-flanking region of five genes (*INHBE*, *PPP1R3B*, *PPP1R3C*, *TUBA4A*, and *GCK*) were designed using Primer3Plus (Supplementary Table S3). PCR products were amplified, verified on 1.5% agarose gel, and enzymatically purified with exonuclease and alkaline phosphatase (Thermo Fisher Scientific, Waltham, MA, USA). Sequencing reactions were prepared with the BigDye Terminator v3.1 Cycle Sequencing Kit (Thermo Fisher Scientific, Waltham, MA, USA), and the resulting fragments were purified using Sephadex G50 (Sigma-Aldrich, St. Louis, MO, USA). Capillary electrophoresis was then executed on an Applied Biosystems Genetic Analyser 3500 (Thermo Fisher Scientific, Waltham, MA, USA), with subsequent sequence alignment and analysis performed via DNASTAR’s SeqMan software (version 12.2.0 (82), 421).

### 2.10. Western Blot

Western blot analysis was conducted on samples obtained from 16 animals in both the E and C groups. Proteins were extracted from liver tissue, with each sample homogenised in radioimmunoprecipitation assay buffer (Sigma-Aldrich, St. Louis, MO, USA). Protein concentration and quality were evaluated using a protein assay kit (Thermo Fisher Scientific, Waltham, MA, USA) on a Qubit fluorometer (Invitrogen, Carlsbad, CA, USA). Due to limited antibody specificity for porcine proteins, only one target protein was ultimately included in the analysis. The primary antibody used was TUBA4A (Abcam, Cambridge, UK; rabbit antibody ab52866-20, dilution 1:8000; 50 kDa). The GAPDH protein (Abcam, Cambridge, UK; mouse antibody ab8245, dilution 1:5000; 36 kDa) was used as the internal reference protein. Protein samples were prepared by combining 25 μg of total protein with Bolt LDS sample buffer and Bolt sample reducing agent (Thermo Fisher Scientific, Waltham, MA, USA). Following denaturation at 70 °C for 10 min, equal volumes of the prepared samples were loaded onto Bolt 12% Bis–Tris Plus gels (Thermo Fisher Scientific, Waltham, MA, USA) for electrophoresis, which was performed at 120 V for 150 min, after which proteins were transferred onto a nitrocellulose membrane (Life Technologies, Carlsbad, CA, USA). Membranes were blocked for 2 h at room temperature using 5% non-fat dry milk prepared in TBST to reduce nonspecific binding. Following blocking, the membranes were washed twice (for 10 min each) with TBST and incubated overnight at 4 °C with the appropriate primary antibodies. After primary antibody incubation, the membranes were washed three times with TBST to remove unbound antibodies and subsequently incubated for 2 h at room temperature with the appropriate horseradish peroxidase (HRP)-conjugated secondary antibody (Abcam, Cambridge, UK): goat anti-rabbit (ab97051, 1:5000) and rabbit anti-mouse (ab6728, 1:5000). Protein bands were visualised using the Immobilon Forte Western HRP substrate (Merck, Darmstadt, Germany) and imaged with a ChemiDoc Touch Imaging System (Bio-Rad Laboratories, Hercules, CA, USA). After the detection of the *TUBA4A* protein, the membranes were washed and stripped with Restore Western Blot Stripping Buffer (Thermo Fisher Scientific, Waltham, MA, USA) for 30 min; washed three times for 5 min with TBS buffer; again blocked with 5% non-fat dry milk for one hour; washed twice for 5 min with TBST; and then incubated overnight at 4 °C with a primary antibody against the reference protein, GAPDH, to normalise *TUBA4A* protein abundance to the loading control. These steps were the same as those used for the *TUBA4A* protein. Band intensities were quantified using Image Lab software, version 1.54g (Bio-Rad Laboratories, Hercules, CA, USA).

### 2.11. Statistical Analyses

Statistical analyses were performed in R version 4.3.1. For RNA-seq data, differential expression analysis was conducted using the DESeq2 model implemented in SeqMonk. *p*-values were adjusted for multiple testing using the Benjamini–Hochberg false-discovery rate (FDR) procedure. Genes with an FDR < 0.05 and passing through the predefined intensity difference filter in SeqMonk were considered differentially expressed. For qPCR and Western blot data, differences were evaluated using the two-tailed Mann–Whitney U test. Pyrosequencing statistical significance was determined based on a two-tailed nonparametric Mann–Whitney U test with FDR correction for each tested CpG locus. Correlations between DNA methylation and gene expression were assessed using Spearman’s rank correlation coefficient. To capture both general trends and phenotype-specific effects, correlations were analysed both across the entire cohort and separately within the E and C groups. FDR-adjusted *p*-values below 0.05 were considered statistically significant. The GO analysis was carried out using the Benjamini–Hochberg method to adjust for multiple testing. GO terms with an adjusted *p*-value (FDR) < 0.05 were considered significantly enriched. Genotype and allele frequencies were calculated for each group. Differences in allele distribution between groups were evaluated by calculating odds ratios (ORs) with 95% confidence using the reference allele as the baseline. The statistical significance of the association was assessed using the Wald test, and *p*-values < 0.05 were considered statistically significant.

## 3. Results

### 3.1. Transcriptomic Changes in Liver Tissue

A total of 169.94 Gb of sequencing data was generated, with an average of 42.2 million fragments per sample (range: 29.7–58.2 million reads). Data quality assessment indicated high read quality (Q30 = 91.92%). Exploratory analyses were also performed, including principal component analysis (PCA) (Supplementary Figure S1).

**Figure 1:**
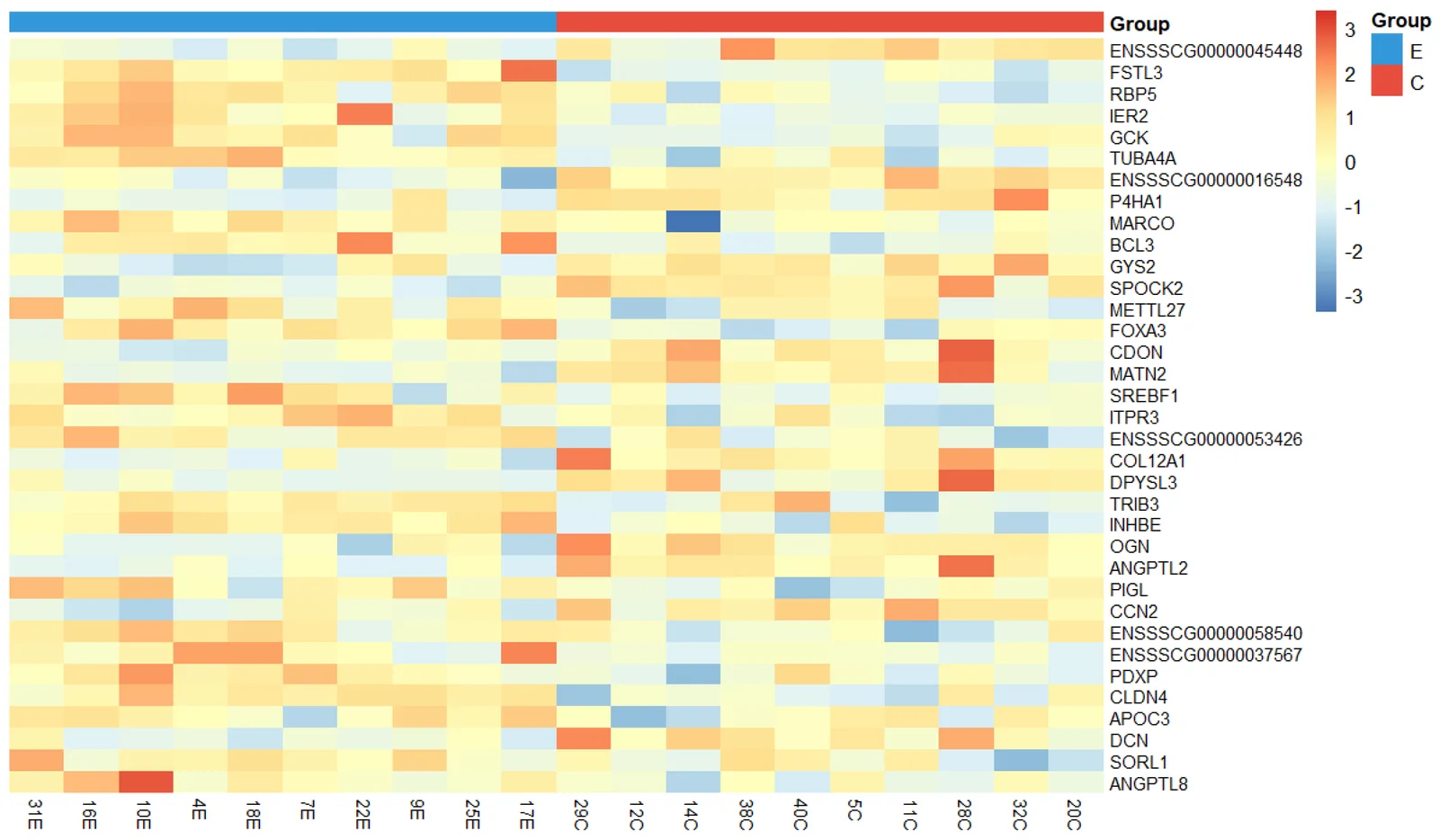
A heatmap of the expression profiles of the top 35 differentially expressed genes ranked according to the SeqMonk differential expression analysis. Gene expression values were variance-stabilised using DESeq2 and standardised by row (z-score) for visualisation. Colours represent relative gene expression levels, where red indicates higher and blue lower expression relative to the mean expression level of each gene across all samples.

In total, 16,103 transcripts were detected and retained for further analysis (Additional Excel File S1). Differential expression analysis identified 104 differentially expressed genes (DEGs) between the maca-supplemented E group and C group based on the predefined threshold of an adjusted *p*-value (FDR) < 0.05. Among these genes, 61 and 43 were up- and downregulated in the E group, respectively. A heatmap presenting the expression profiles of the top 35 DEGs ranked according to the SeqMonk analysis is shown in Figure 1. The raw sequencing data were deposited in the NCBI Sequence Read Archive under accession number BioProject ID: PRJNA1451424.

### 3.2. qPCR Validation of RNA-Seq Results

**Figure 2:**
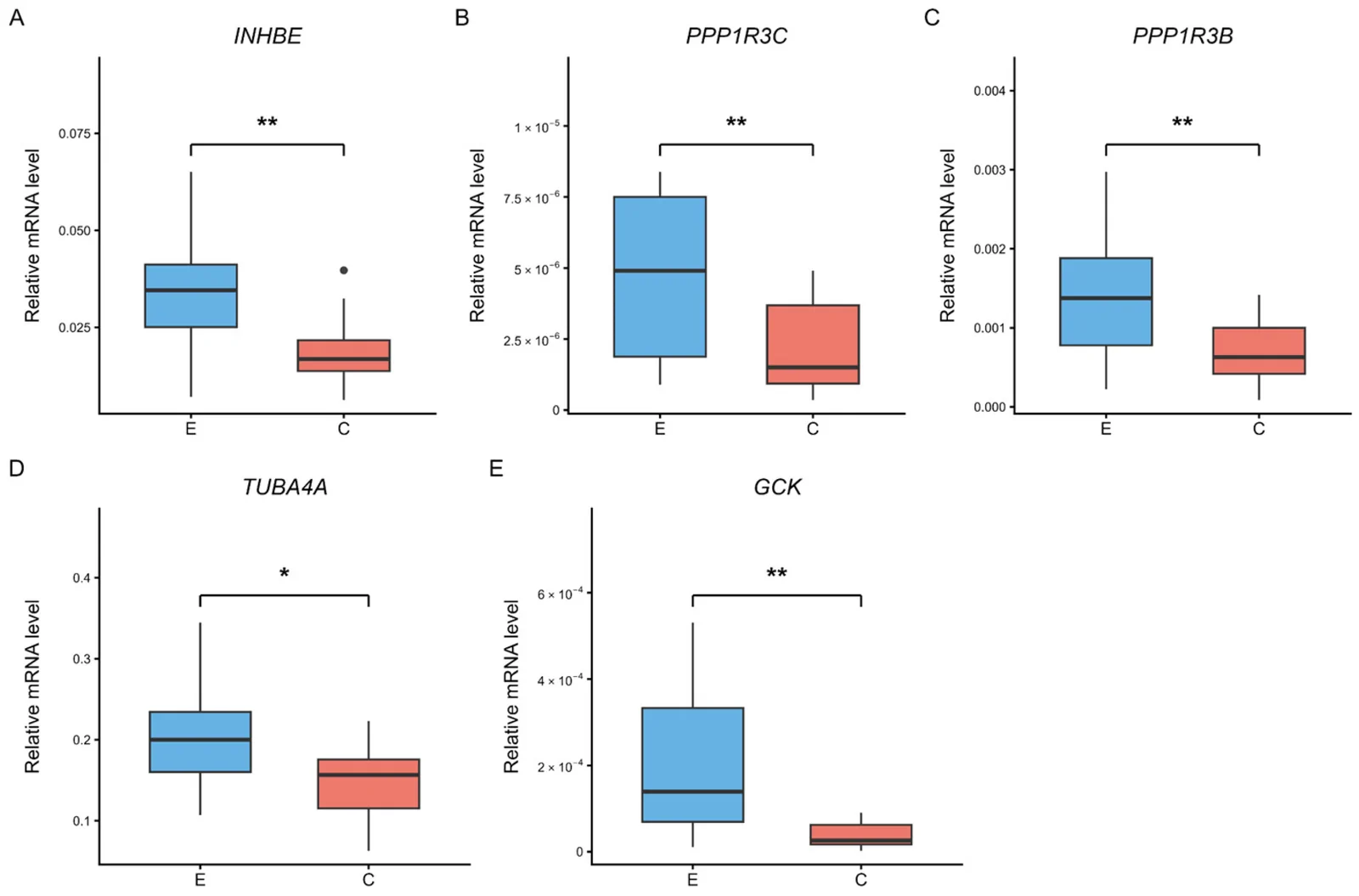
Relative mRNA expression levels of the selected genes. Box plots showing the relative expression levels of the (**A**) *INHBE*, (**B**) *PPP1R3C*, (**C**) *PPP1R3B*, (**D**) *TUBA4A*, and (**E**) *GCK* genes determined using RT-qPCR in the experimental group ((**E**), n = 16) compared to the control group ((**C**), n = 16). The horizontal line inside the box indicates the median, the box boundaries represent the interquartile range (IQR), whiskers show the data range within 1.5 times the IQR, and the black dot indicates a statistical outlier. * *p*-value < 0.05; ** *p*-value < 0.01.

The qPCR analysis confirmed statistically significant differential expression for five of the nine genes examined (Table 1). Specifically, *INHBE*, *PPP1R3C*, *PPP1R3B*, *TUBA4A*, and *GCK* (with *p*-values of 0.0013, 0.0033, 0.0088, 0.0168, and 0.0040, respectively) were all significantly upregulated in the E group compared with the C group, as shown in Figure 2. For the four genes that did not reach statistical significance based on qPCR (*SREBF1*, *DCN*, *IGFALS*, and *CLDN4*), the direction of expression changes was consistent with the RNA-seq results. Notably, *SREBF1* showed a trend toward statistical significance (*p* = 0.0574).

### 3.3. Analysis of Functional Enrichment Pathway

**Figure 3:**
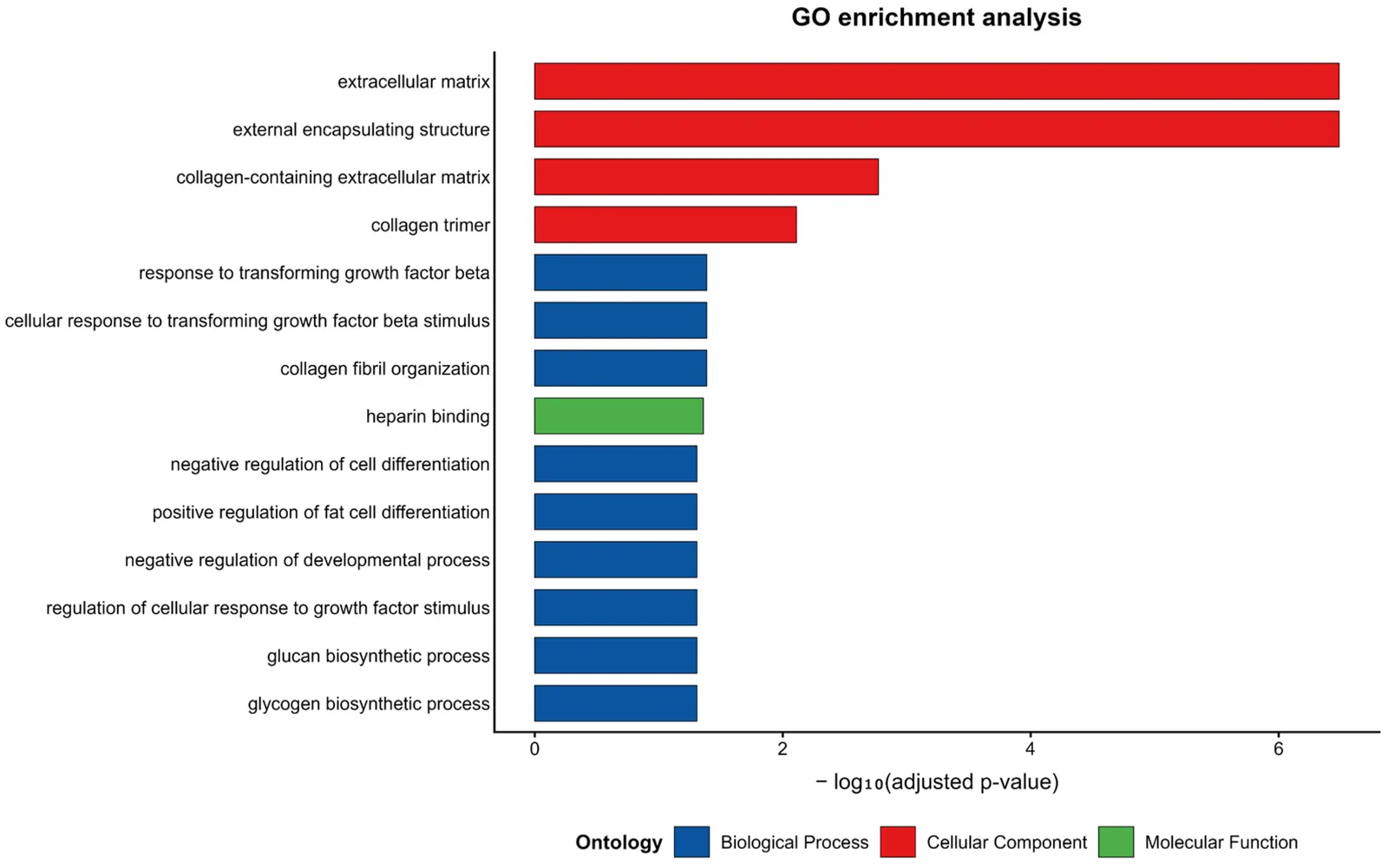
Gene Ontology (GO) enrichment analysis of differentially expressed genes. The bar plot shows significantly enriched GO terms ranked according to the −log_10_-transformed adjusted *p*-values. Colours represent the three GO ontologies: Biological Process (blue), Cellular Component (red), and Molecular Function (green). Adjusted *p*-values were calculated using the Benjamini–Hochberg FDR method.

The GO enrichment analysis revealed significant enrichment across all three GO domains. Biological Process terms were mainly associated with collagen fibril organisation, transforming growth factor beta response, glycogen biosynthesis, and the regulation of cell differentiation. Molecular Function analysis identified heparin binding as significantly enriched. Cellular Component terms showed the strongest enrichment and were primarily related to extracellular matrix structures, including the external encapsulating structure, extracellular matrix, collagen-containing extracellular matrix, and collagen trimer, as shown in Figure 3 and Additional Excel File S2.

### 3.4. DNA Methylation Analysis Using Pyrosequencing

**Figure 4:**
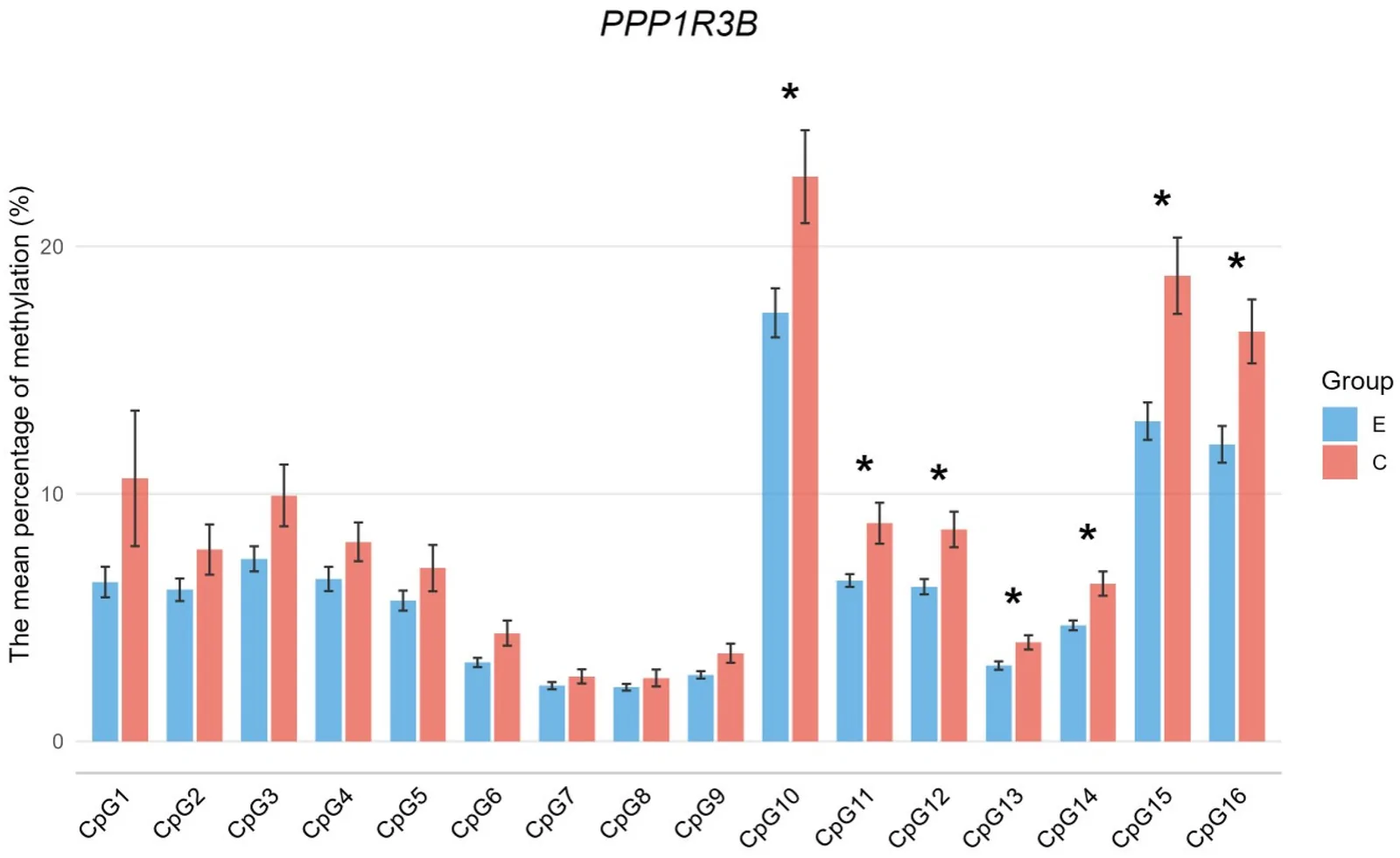
DNA methylation analysis of CpG sites in *PPP1R3B* determined using pyrosequencing. Bars represent mean methylation percentages ± SD in the E (blue) and C (red) groups. Significant differences between groups at individual CpG sites are indicated by asterisks: * FDR-adjusted *p* < 0.05.

The analysis of five CpG sites within the *GCK* gene locus revealed no statistically significant differences in DNA methylation levels between the E and C groups after multiple testing correction (FDR > 0.05). Similarly, none of the four evaluated CpG sites within the *INHBE* gene remained statistically significant after Benjamini–Hochberg correction (FDR > 0.05). The methylation analysis of the four monitored CpG sites in *PPP1R3C* revealed a highly stable and homogeneous pattern across both experimental groups, while the six analysed CpG sites within *TUBA4A* also exhibited comparable methylation levels between groups. The results are presented in Supplementary Figure S2. In contrast to the other genes, the *PPP1R3B* gene displayed significant differences in DNA methylation that successfully withstood the multi-test correction. Among the 16 analysed CpG sites, 7 sites showed statistically significant differences between the groups (FDR < 0.05). Specifically, the most pronounced differential methylation profiles were confirmed at CpG12 (FDR = 0.0112), CpG14 (FDR = 0.0112), CpG15 (FDR = 0.0112), and CpG16 (FDR = 0.0153). Furthermore, significant experimental effects were validated at CpG11 (FDR = 0.0238), as well as CpG10 and CpG13 (both sharing FDR = 0.0408). The remaining sites (CpG1–CpG9) did not show significant group differences after correction, as shown in Figure 4.

Spearman correlation analysis did not reveal any significant associations between CpG methylation levels and gene expression after correction for multiple testing (FDR > 0.05). Although several nominal correlations (*p* < 0.05) were observed, including negative correlations for *INHBE* and positive ones for *GCK* and *TUBA4A*, none remained significant after Benjamini–Hochberg correction. Additionally, in terms of *PPP1R3B*, in which seven CpG sites had significantly a lower methylation level than that in the E group, the mRNA level was significantly enhanced, and the correlation analysis showed no statistical significance after FDR correction (Supplementary Table S4).

### 3.5. Sanger Sequencing

**Figure 5:**
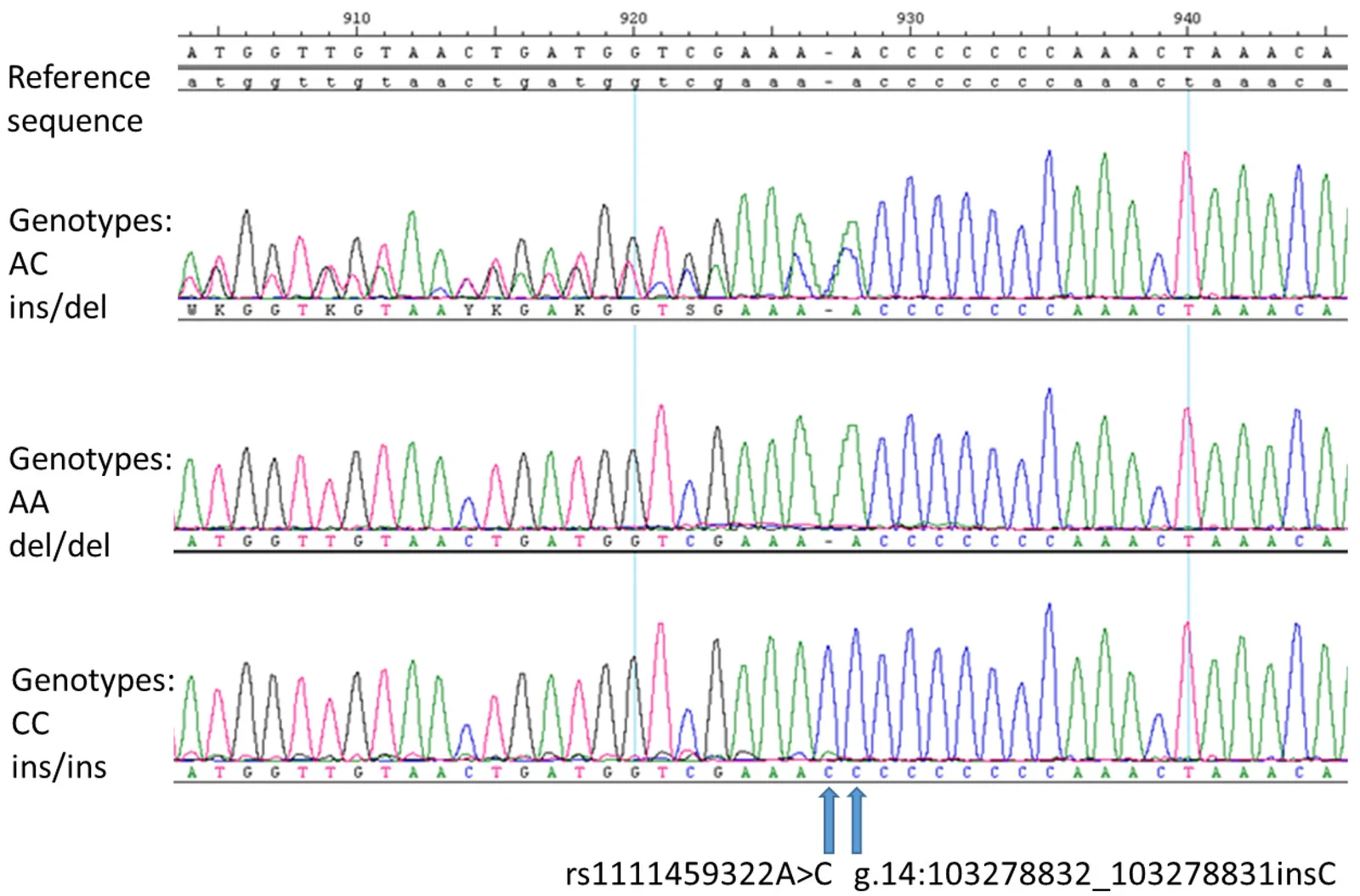
Sanger sequencing chromatograms showing the promoter polymorphisms identified in the *PPP1R3C* gene. The chromatograms illustrate the three observed genotype combinations: AA; del/del, AC; ins/del, and CC; ins/ins.

In the *PPR1R3C* gene, two variants were found, A > C (rs 1111459322) substitution and the C insertion (g.14:103278832_103278831insC), which presented complete co-segregation, as shown in Figure 5. Consequently, the statistical results obtained for both variants were identical, and thus the frequencies of the AA, AC, and CC genotypes corresponded exactly to those of the del/del, ins/del, and ins/ins genotypes, respectively. In the E group, the frequencies of the AA; del/del, AC; ins/del, and CC; ins/ins genotypes were 60.0% (n = 12), 35.0% (n = 7), and 5.0% (n = 1), respectively. In the C group, the genotype distribution was 50.0% (n = 10) for AA; del/del, 45.0% (n = 9) for AC; ins/del, and 5.0% (n = 1) for CC; ins/ins. No significant differences in genotype distribution were observed between the E and C groups (odds ratio = 0.7654; 95% CI = 0.2771 to 2.1143; *p*-value = 0.6060). To assess the potential functional relevance of *PPP1R3C* promoter polymorphism, *PPP1R3C* expression levels were compared between the AA and AC genotypes. No significant differences in gene expression were observed between genotypes in the control (*p*-value = 0.9912) or maca-supplemented group (*p*-value = 0.3310) or when both treatment groups were analysed together (*p*-value = 0.9825) (Supplementary Table S5). The CC genotype was not included in the statistical comparison because of its very low frequency.

For the *TUBA4A* gene, only two genotypes were identified, GG and GA (rs345885565, G > A), as shown in Supplementary Figure S3. A single heterozygous (GA) individual was detected in both the E and C groups, whereas all remaining animals were homozygous for the reference allele (GG). No AA homozygotes were observed. Consequently, the minor allele occurred at a very low frequency in the analysed population.

### 3.6. Western Blot Analysis

The results showed no significant difference in TUBA4A protein levels between the E and C groups, indicating comparable protein levels across all analysed samples (Supplementary Figure S4).

## 4. Discussion

The present study provides evidence that dietary supplementation with *L. meyenii* is associated with measurable hepatic molecular responses in pigs. The differential expression analysis identified 104 DEGs. This suggests that the effect of maca supplementation on the liver was moderate rather than strongly group-separating, but it was still detectable at the level of specific genes and biological processes. Similar observations have been reported in nutrigenomic studies in pigs, in which dietary interventions modulated selected metabolic pathways and differentially expressed genes without causing extensive global transcriptomic remodelling [21,22]. qPCR validation showed partial concordance with the RNA-seq results, with five of the nine selected genes reaching statistical significance. Importantly, the rest of the studied genes showed expression changes in the same direction as observed using RNA-seq, although these differences did not reach statistical significance based on qPCR. 

Experimental studies have demonstrated that hepatic *PPP1R3B* regulates glycogen storage and influences circulating glucose concentrations [12]. In the present study, the observed increase in *PPP1R3B* expression may be relevant to the altered glucose homeostasis observed in maca-supplemented pigs. *PPP1R3B* encodes a glycogen-targeting regulatory subunit of protein phosphatase 1 (PP1) that plays an important role in hepatic glycogen synthesis and glucose homeostasis. The increased *PPP1R3B* expression level, accompanied by an enrichment in glycogen biosynthetic processes, is supported by the higher blood glucose concentrations seen in maca-supplemented pigs, which were also observed in our earlier study [10]. Together, these findings are consistent with the potential involvement of the *PPP1R3B*-associated regulation of hepatic carbohydrate metabolism in the response to maca supplementation. However, as hepatic glycogen content, glycogen synthase activity, and *PPP1R3B* protein abundance were not determined, a direct functional link between the observed transcriptional changes and glucose homeostasis cannot be established.

However, this interpretation is further supported by the differential expression of *GCK*, which encodes glucokinase, a key enzyme controlling hepatic glucose phosphorylation and glycogen synthesis after feeding. Nutritional interventions have previously been shown to modulate hepatic glucose metabolism in pigs. For example, Ying et al. demonstrated that long-term dietary methionine restriction (30% reduction in methionine from weaning until 180 days of age) in pigs with intrauterine growth restriction increased hepatic *GCK* expression, enhanced hepatic glycogen synthesis, and reduced plasma glucose concentration. These findings show that dietary factors can regulate the hepatic pathways involved in glucose utilisation and glycogen storage [23]. *INHBE* was also significantly affected by the dietary treatment. This gene encodes activin E, a liver-derived hepatokine belonging to the transforming growth factor beta (TGF-β) superfamily. A recent study by Adam et al. [24] identified activin E as an important mediator of liver–adipose tissue communication that regulates energy storage by suppressing adipose tissue lipolysis through ACVR1C-SMAD signalling. This limits the mobilisation of stored triglycerides into circulating free fatty acids and glycerol and contributes to systemic insulin sensitivity [24]. Park et al. [25] demonstrated that activin E also protects against hepatic steatosis. In mice, activin E deficiency increased adipose lipolysis, thus resulting in greater fatty acid influx into the liver, hepatic triglyceride accumulation, and hepatic insulin resistance. Meanwhile, the hepatic overexpression of *INHBE* suppressed adipose lipolysis and reduced liver fat accumulation, thereby highlighting its protective role in hepatic lipid homeostasis [25]. Therefore, the change in *INHBE* expression observed here may reflect a broader endocrine response of the liver to maca supplementation. However, since circulating activin E, insulin, and detailed lipid flux markers were not measured in this study, this interpretation should be treated as a hypothesis, not a conclusion. The hepatic transcriptional changes observed in the present study may be related to the diverse bioactive constituents of maca, which include macamides, macaenes, glucosinolates, polyphenols, and other secondary metabolites [6]. These compounds have been associated with antioxidant, metabolic, anti-inflammatory, and other regulatory activities, thus suggesting that maca may influence cellular processes through the combined effects of several phytochemical classes [6]. In the present study, the simultaneous upregulation of *GCK*, *PPP1R3B*, and *PPP1R3C*, together with the enrichment in glycogen biosynthetic processes, suggests that maca supplementation may influence hepatic glucose utilisation and energy storage. Macamides, macaenes, glucosinolates, and polyphenols may contribute to these effects through their reported metabolic, regulatory, antioxidant, and redox-modulating activities [6]. Thus, the combined activity of maca phytochemicals may contribute to the hepatic transcriptional response observed in the present study.

The GO enrichment analysis revealed several related and partially overlapping GO terms that collectively indicated an enrichment in processes associated with the extracellular matrix and collagen organisation. These pathways are commonly associated with tissue structure and remodelling, and increasing evidence indicates that they also contribute to the regulation of liver function and metabolism [26,27]. The hepatic extracellular matrix is now recognised as a dynamic component that interacts with hepatocytes and other liver cells, which influences cell signalling, tissue homeostasis, and metabolic adaptation. TGF-β signalling plays an important role in maintaining liver homeostasis and has been implicated in the regulation of insulin sensitivity and hepatic glucose metabolism [28]. Therefore, the enrichment in these pathways may reflect the metabolic adaptation of the liver to dietary maca supplementation. This interpretation is further supported by qPCR validation, which confirmed the altered expression of genes involved in glycogen and glucose metabolism, including *PPP1R3B*, *PPP1R3C*, and *GCK*. Consistent with the present findings, Oczkowicz et al. [29] demonstrated that different dietary fat sources (rapeseed oil, beef tallow, and coconut oil) markedly altered the porcine liver transcriptome, thus affecting pathways related to cholesterol metabolism, bile acid biosynthesis, unfolded protein response, immune signalling, and cellular metabolism. These observations further support the high responsiveness of the liver transcriptome to dietary interventions. The targeted DNA methylation analysis provided an additional regulatory layer. Most analysed genes, including *GCK*, *INHBE*, *PPP1R3C*, and *TUBA4A*, did not show significant differences in promoter CpG methylation. In contrast, *PPP1R3B* showed significant differences in DNA methylation at seven CpG sites; however, no significant correlations between methylation levels and gene expression remained after FDR correction. This canonical negative correlation between promoter DNA methylation and mRNA level is a well-known epigenetic mechanism responsible for the tissue-specific gene expression profile [30]. However, it must be noted that such observations were only obtained for one gene here. Promoter regions are key elements that control gene transcription. They coordinate the recruitment of RNA polymerase and transcription factors to the transcription start site. Even single-nucleotide substitutions or short insertions/deletions within promoter sequences may alter the affinity of transcription factor binding sites, thereby modifying transcriptional activity and ultimately affecting gene expression. Studies demonstrated that promoter polymorphisms may contribute to variation in gene expression and the tendency to display phenotypic differences by influencing the binding of regulatory proteins [31,32]. To investigate whether the identified promoter variants could explain the altered hepatic expression observed, we searched for DNA variants in five genes that showed RNA-level abundance. Only three variants (two in *PPP1R3C*) and one with a very low alternative allele frequency in *TUBA4A* genes were identified. The two co-segregating variants in *PPP1R3C* did not differ in allele frequencies between studied groups; thus, the identified promoter polymorphisms are unlikely to account for the changed hepatic *PPP1R3C* expression observed after maca supplementation. The lack of significant differences in *TUBA4A* protein abundance despite altered mRNA expression highlights the importance of multi-level validation. Discordance between transcript and protein abundance may result from post-transcriptional regulation, differences in mRNA and protein stability, translational control, or the limited sensitivity of antibody-based detection [33,34]. Thus, the Western blot results do not invalidate the RNA-level findings, but they do indicate that the transcriptional change was not reflected in the detectable protein abundance under the conditions tested. The present study also has several limitations. First, the relatively small number of animals, particularly in the Sanger sequencing analysis, reduced the statistical power to detect genotype-dependent differences in gene expression. The low frequency of some promoter variants (the CC; ins/ins genotype of *PPP1R3C*) further limited genotype-based comparisons. Moreover, this study was conducted in a single commercial population of female pigs (gilts), and therefore the findings should be verified in larger populations, different breeds, and both sexes. Finally, protein-level validation was limited by the restricted availability of highly specific antibodies against porcine proteins, thus preventing the verification of all candidate genes identified using transcriptomic analysis.

## 5. Conclusions

The present study demonstrated that dietary supplementation with 1% maca was associated with hepatic molecular changes in growing pigs, including the differential expression of *INHBE*, *PPP1R3C*, *PPP1R3B*, *TUBA4A*, and *GCK*. These changes were accompanied by enrichment in glycogen biosynthetic and extracellular matrix-related processes, while *PPP1R3B* showed treatment-associated methylation differences at seven CpG sites. Although the functional consequences of these molecular changes remain to be determined, we consider *L. meyenii* a promising natural feed supplement rather than a validated feeding strategy. Adequately replicated studies under commercial production conditions are needed to confirm its efficacy, determine its optimal dose, and assess the consistency and long-term practical benefits of supplementation.

## Figures and Tables

**Table 1:** RNA-seq and RT-qPCR results for 9 selected genes.

Gene Symbol	RNA-seq		RT-qPCR	
	log_2_FC	padj	log_2_FC	*p*-Value
*GCK*	2.349	0.0002	2.280	0.0040
*TUBA4A*	0.999	0.0005	0.427	0.0168
*SREBF1*	1.118	0.0027	0.971	0.0574
*INHBE*	1.159	0.0034	0.849	0.0013
*CLDN4*	1.036	0.0056	0.044	0.9283
*DCN*	−1.151	0.0067	−0.578	0.0929
*PPP1R3C*	1.097	0.0206	1.122	0.0033
*PPP1R3B*	1.052	0.0277	1.052	0.0088
*IGFALS*	1.129	0.0009	0.480	0.1362

## Data Availability

The raw sequencing data were deposited in the NCBI Sequence Read Archive under accession number BioProject ID: PRJNA1451424. The dataset generated and analysed during the present study was deposited in an official data repository (https://repod.icm.edu.pl/dataset.xhtml?persistentId=doi:10.18150/BKSUGD (accessed on 29 May 2026)).

## Supplementary Materials

The following supporting information can be downloaded at https://www.mdpi.com/article/10.3390/genes17091128/s1, Table S1: Primer sequences for *CLDN4*, *IGFALS*, *INHBE*, *DCN*, *SREBF1*, *PPP1R3C*, *PPP1R3B*, *TUBA4A*, *GCK*, *H3F3A*, and *PPIA* genes used in qPCR analysis; Table S2: Primers for *INHBE*, *PPP1R3C*, *PPP1R3B*, *TUBA4A*, and *GCK* genes used in pyrosequencing; Table S3: Primers for *INHBE*, *PPP1R3C*, *PPP1R3B*, *TUBA4A*, and *GCK* genes used in Sanger sequencing; Table S4: Correlations between DNA methylation at individual CpG sites determined using pyrosequencing and relative gene expression levels studied via RT-qPCR; Table S5: *PPP1R3C* relative expression levels according to *PPP1R3C* promoter genotype; Figure S1: RNA-seq data visualisation—principal component analysis (PCA) based on variance-stabilised transformed (VST) expression data; Figure S2: DNA methylation profiles of *INHBE*, *PPP1R3C*, *TUBA4A*, and *GCK* genes determined using pyrosequencing; Figure S3: Sanger sequencing results for *TUBA4A* gene; Figure S4: Relative TUBA4A protein expression level; Additional Excel File S1: A list of DEGs obtained from RNA-seq analysis; Additional Excel File S2: A list of GO processes obtained from Gene Ontology analysis.

## Abbreviations

The following abbreviations are used in this manuscript.

RNA-seq	RNA sequencing
DEGs	differently expressed genes
qPCR	quantitative PCR
HRP	horseradish peroxidase
CpG sites	cytosine–phosphate–guanine sites
GO	Gene Ontology
RIN	RNA integrity number
FDR	false-discovery rate
insC	cytosine insertion
PP1	protein phosphatase 1
TGF-β	transforming growth factor beta
PCA	principal component analysis
VST	variance-stabilised transformed
