# Supplementary material for: Dietary Lepidium meyenii Supplementation Is Associated with Hepatic Transcriptomic and Targeted DNA Methylation Changes in Pigs
Source: Genes (Basel). 2026 Sep 16;17(9):1128. doi: 10.3390/genes17091128 (PMC13606173; doi:10.3390/genes17091128)
Supplement: Supplementary file 1 [file genes-17-01128-s001.zip › genes-4531257-supplementary.pdf]

Table S1: Primer sequences for *CLDN4, IGFALS, INHBE, DCN, SREBF1, PPP1R3C, PPP1R3B, TUBA4A, GCK, H3F3A*, and *PPIA* genes used in qPCR analysis

Gene	Sequence	Product length (bp)
CLDN4	L: GCTAGCCCTGTTGCTCAGTT	314
	R: GATACCCATCACCTGCAGCC	
IGFALS	L: GATGGCCCTGAGGAAAGGAG	171
	R: CTGAGCTCGTCCGTGTAGTC	
INHBE	L: AGGAGGTCATCAGCTTTGCT	150
	R: AAAGAGTGCCAGGAAGGGTG	
DCN	L: CTACATCCGCATCGCTGACA	116
	R: TTAGGCTAGCTGCATCAACTT	
SREBF1	L: CCTGTCTGGGGCTCTCCT	139
	R: CAGCGTCTGTGAGGTGCTC	
PPP1R3C	L: CGTGGTCGGGAGCAGATAAG	184
	R: AACTTGTCAAAGGCCGTGGA	
PPP1R3B	L: ATGCGACTGTCAGCGGAG	182
	R: TCAATGTCCACAGCCATCAC	
TUBA4A	L: CCGGGCAGTTTTTGTGGATT	134
	R: AGTGACCACGGGCATAGTTG	
GCK	L: TGAAGCACAAAAAGCTGCCC	157
	R: CGTTTGATGGCATCTCGCAG	
Reference genes		
H3F3A	F: CTTTGCAGGAGGCAAGTGAG	72
	R: TGGCATGGATAGCACACAGG	
PPIA	F: CACAAACGGTTCCCAGTTTT	171
	R: TGTCCACAGTCAGCAATGGT	

Table S2: Primers for *INHBE, PPP1R3C, PPP1R3B, TUBA4A*, and *GCK* genes used in pyrosequencing

Gene (strand location in the genome)	Sequence	Product length (bp)	Number of analysed cytosines	Genomic position of first and last analysed cytosine
INHBE **(forward)**	F: 5′ TTGGGGATTGAGTATGTGAAATTA—biot<br>R: 5′ CATATCTAACTACTAACTAAAACCTCTCTA<br>S: 5′ CCTCTCTAAAACCAACA	271	CpG1–CpG4	Chr5: 22,732,510–22,732,382
PPP1R3C **(reverse)**	F: 5′ AGAGGATTATTGGATAATTAAGAGAGTAG<br>R: 5′ AATCAATCCAAATCCCTCAATTCTAT—biot<br>S: 5′ AGGATAGGGGTTTTATAAAAAT	257	CpG1–CpG4	Chr14: 103,278,875–103,278,764
PPP1R3B **(forward)**	F: 5′ GGGAGAGGGAATTTTGTTAGTTA<br>R: 5′ TCTCCCCACTAAAAATACCATCCC–biot<br>S: 5′ AGGTTTTTGATGAAATAGTGATTTA	314	CpG1–CpG16	Chr15: 56,002,204–56,002,329
TUBA4A **(reverse)**	F: 5′ TAGGGATAGAAGAGGATGGTAAAA<br>R: 5′ CCTACTAAAACCCTATATAACCTTAAAC–biot<br>S: 5′ TTTTTTTTAGAGTTAATAAATATTG	206	CpG1 - CpG6	Chr15: 121,294,809–121,294,750
GCK **(forward)**	F: 5′ ATAGGAAGTTGGTTTAAAAATGTTATAGT–biot<br>R: 5′ ACCTAAAAAATAAAAACCCCATAAATC<br>S: 5′ CAACATTACCACATATACC	210	CpG1 - CpG5	Chr18: 50,978,928–50,978,855

Genomic position according to Sscrofa11 assembly.

Table S3: Primers for *INHBE, PPP1R3C, PPP1R3B, TUBA4A*, and *GCK* genes used in Sanger sequencing

Gene	Sequence	Primer's genomic position	Product length (bp)
INHBE	F: TCATGCGTGTCCACATAGCC<br>R: GACCAGAGCTCGTTCTGCTT	Chr5<br>F: 22,732,332–22,732,351<br>R: 22,732,907–22,732,926	595
PPP1R3C	F: TTTTGGTTGGGCTGAGAGGC<br>R: CCCTGGAAGAGAAAACTAAAGCC	Chr14<br>F: 103,279,132–103,279,151<br>R: 103,278,606–103,278,628	546
PPP1R3B	F: TGTAGGGTGACTTTGCCACG<br>R: TGGCAGGGGCAACGGTAG	Chr15:<br>F: 56,002,045–56,002,064<br>R: 56,002,552–56,002,569	525
TUBA4A	F: TTCTCCCAACCACATGGCAA<br>R: GAGGAGACAAGATTCCGCCC	Chr15:<br>F: 121,295,350–121,295,369<br>R: 121,294,776–121,294,795	594
GCK	F: ACGTGCACAGCATCTGATCT<br>R: ATTCTCCTGCCAGGGCTTTC	Chr18:<br>F: 50,978,667–50,978,686<br>R: 50,979,247–50,979,266	600

Genomic position according to Sscrofa11 assembly.

Table S4: Correlations between DNA methylation at individual CpG sites determined using pyrosequencing and relative gene expression levels studied via RT-qPCR

Gene	Analysis type	CpG site	Cor	P-value	Adjusted P-value
INHBE	Global	CpG1	-0.3	0.091	0.121
	Global	CpG2	-0.38	0.034	0.067
	Global	CpG3	-0.46	0.009	**0.035**
	Global	CpG4	-0.12	0.507	0.507
	Group E	CpG1	-0.19	0.484	0.645
	Group C	CpG1	-0.29	0.279	0.372

	Group E	CpG2	-0.21	0.427	0.645
	Group C	CpG2	-0.49	0.055	0.220
	Group E	CpG3	-0.33	0.208	0.645
	Group C	CpG3	-0.32	0.226	0.372
	Group E	CpG4	-0.099	0.716	0.716
	Group C	CpG4	-0.17	0.533	0.533
GCK	Global	CpG1	0.15	0.422	0.703
	Global	CpG2	0.39	0.029	0.143
	Global	CpG3	0.02	0.913	0.913
	Global	CpG4	0.19	0.297	0.703
	Global	CpG5	-0.065	0.725	0.906
	Group E	CpG1	0.26	0.323	0.461
	Group C	CpG1	-0.28	0.290	0.395
	Group E	CpG2	0.62	0.010	0.052
	Group C	CpG2	-0.16	0.559	0.559
	Group E	CpG3	-0.0074	0.978	0.978
	Group C	CpG3	-0.27	0.316	0.395
	Group E	CpG4	0.27	0.312	0.461
	Group C	CpG4	-0.52	0.037	0.186
	Group E	CpG5	-0.24	0.369	0.461
	Group C	CpG5	-0.45	0.083	0.207
TUBA4A	Global	CpG1	0.18	0.319	0.485
	Global	CpG2	0.25	0.168	0.485
	Global	CpG3	0.08	0.662	0.662
	Global	CpG4	0.15	0.404	0.485
	Global	CpG5	0.17	0.367	0.485
	Global	CpG6	0.42	0.017	0.100
	Group E	CpG1	0.18	0.496	0.744
	Group C	CpG1	0.31	0.247	0.748

	Group E	CpG2	0.25	0.344	0.744
	Group C	CpG2	0.17	0.520	0.748
	Group E	CpG3	0.032	0.905	0.905
	Group C	CpG3	0.13	0.639	0.748
	Group E	CpG4	0.11	0.688	0.826
	Group C	CpG4	0.087	0.748	0.748
	Group E	CpG5	0.2	0.450	0.744
	Group C	CpG5	0.18	0.502	0.748
	Group E	CpG6	0.56	0.024	0.143
	Group C	CpG6	0.19	0.491	0.748
PPP1R3C	Global	CpG1	-0.24	0.194	0.388
	Global	CpG2	-0.18	0.338	0.451
	Global	CpG3	-0.28	0.134	0.388
	Global	CpG4	-0.13	0.483	0.483
	Group E	CpG1	0.24	0.390	0.954
	Group C	CpG1	-0.38	0.147	0.338
	Group E	CpG2	0.16	0.575	0.954
	Group C	CpG2	-0.015	0.957	0.957
	Group E	CpG3	0.016	0.954	0.954
	Group C	CpG3	-0.36	0.169	0.338
	Group E	CpG4	0.045	0.874	0.954
	Group C	CpG4	-0.24	0.367	0.489
PPP1R3B	Global	CpG1	-0.072	0.695	0.881
	Global	CpG2	-0.029	0.876	0.881
	Global	CpG3	-0.16	0.387	0.563
	Global	CpG4	-0.028	0.881	0.881
	Global	CpG5	0.033	0.859	0.881
	Global	CpG6	-0.25	0.165	0.382
	Global	CpG7	-0.063	0.732	0.881

Global	CpG8	-0.17	0.346	0.554
Global	CpG9	-0.18	0.317	0.554
Global	CpG10	-0.23	0.212	0.424
Global	CpG11	-0.27	0.141	0.382
Global	CpG12	-0.3	0.094	0.382
Global	CpG13	-0.44	0.012	0.195
Global	CpG14	-0.3	0.099	0.382
Global	CpG15	-0.25	0.167	0.382
Global	CpG16	-0.35	0.052	0.382
Group E	CpG1	0.071	0.795	0.984
Group C	CpG1	0.059	0.829	0.996
Group E	CpG2	0.049	0.857	0.984
Group C	CpG2	0.08	0.769	0.996
Group E	CpG3	0.29	0.284	0.984
Group C	CpG3	-0.23	0.397	0.996
Group E	CpG4	0.3	0.257	0.984
Group C	CpG4	0.021	0.939	0.996
Group E	CpG5	0.28	0.297	0.984
Group C	CpG5	-0.0015	0.996	0.996
Group E	CpG6	-0.21	0.437	0.984
Group C	CpG6	0.15	0.573	0.996
Group E	CpG7	-0.0086	0.975	0.984
Group C	CpG7	0.095	0.726	0.996
Group E	CpG8	0.0054	0.984	0.984
Group C	CpG8	-0.32	0.226	0.996
Group E	CpG9	-0.1	0.711	0.984
Group C	CpG9	0.11	0.694	0.996
Group E	CpG10	0.038	0.888	0.984
Group C	CpG10	-0.047	0.862	0.996

Group E	CpG11	-0.052	0.850	0.984
Group C	CpG11	-0.01	0.969	0.996
Group E	CpG12	-0.078	0.774	0.984
Group C	CpG12	-0.011	0.969	0.996
Group E	CpG13	-0.41	0.118	0.984
Group C	CpG13	-0.1	0.703	0.996
Group E	CpG14	-0.016	0.953	0.984
Group C	CpG14	0.069	0.799	0.996
Group E	CpG15	0.2	0.457	0.984
Group C	CpG15	0.039	0.887	0.996
Group E	CpG16	0.056	0.836	0.984
Group C	CpG16	-0.23	0.398	0.996

Correlation analyses were performed for all samples combined (Global), the experimental group (E), and the control group (C). The table presents the correlation (Cor), nominal *p*-values, and Benjamini–Hochberg-adjusted *p*-values (FDR) for each CpG site. Groups: control (Group C) and maca-supplemented with maca (Group E).

Table S5. *PPP1R3C* relative expression levels according to *PPP1R3C* promoter genotype.

**Comparison**	**P-value**
AA vs AC–C group	0.9912
AA vs AC–E group	0.3310
AA vs AC–all animals	0.9825

No significant differences in expression were detected between genotypes. The CC genotype was excluded from statistical comparisons because of its low frequency.

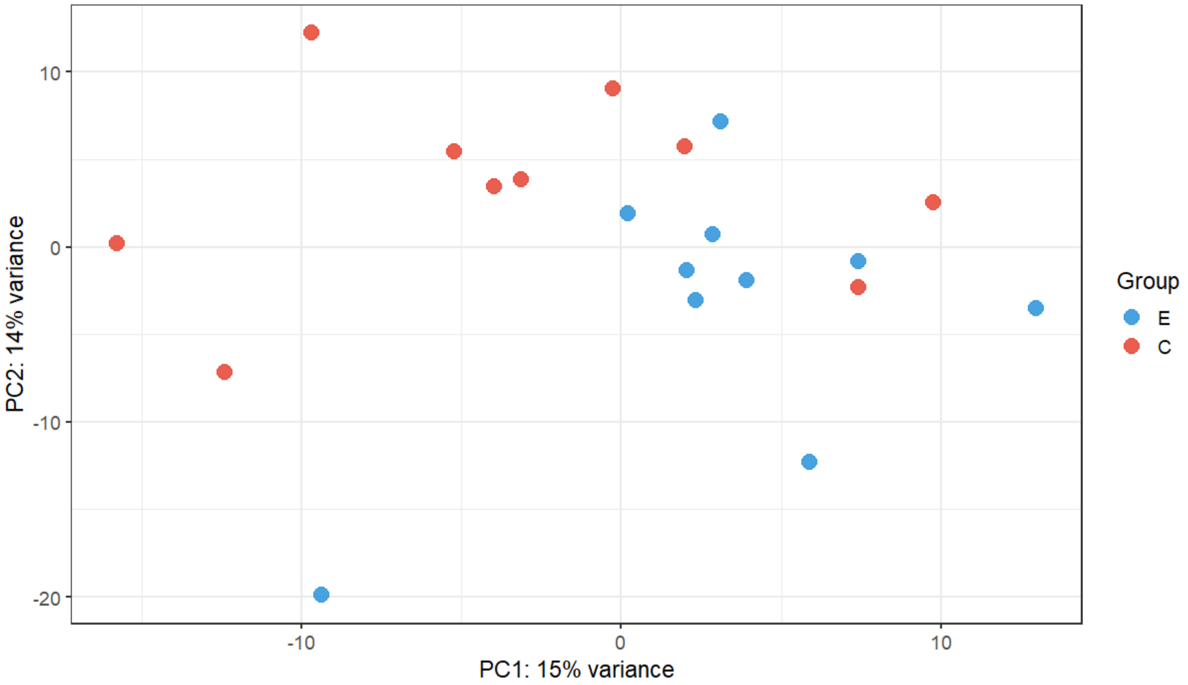

Figure S1: RNA-seq data visualisation—principal component analysis (PCA) based on variance-stabilised transformed (VST) expression data. Samples are displayed according to the first two principal components (PC1 and PC2), which explained 15% and 14% of the total variance, respectively. Samples are coloured according to the studied groups: maca-supplemented (E) blue and control (C) red.

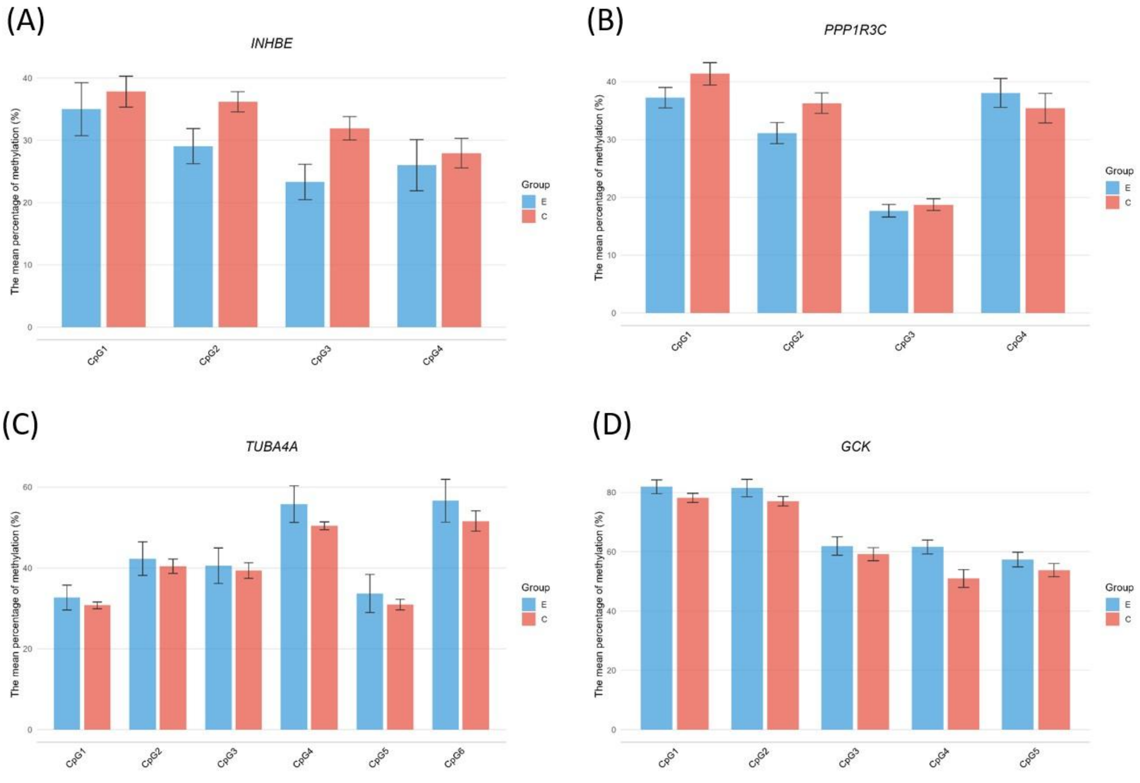

Figure S2. DNA methylation profiles of *INHBE, PPP1R3C, TUBA4A*, and *GCK* genes determined using pyrosequencing. Mean DNA methylation (%) at individual CpG sites within (A) *INHBE*, (B) *PPP1R3C*, (C) *TUBA4A*, and (D) *GCK* in the experimental (E—blue) and control (C—red) groups. Bars represent mean ± SD. None of the analysed CpG sites remained significantly different between groups after FDR correction.

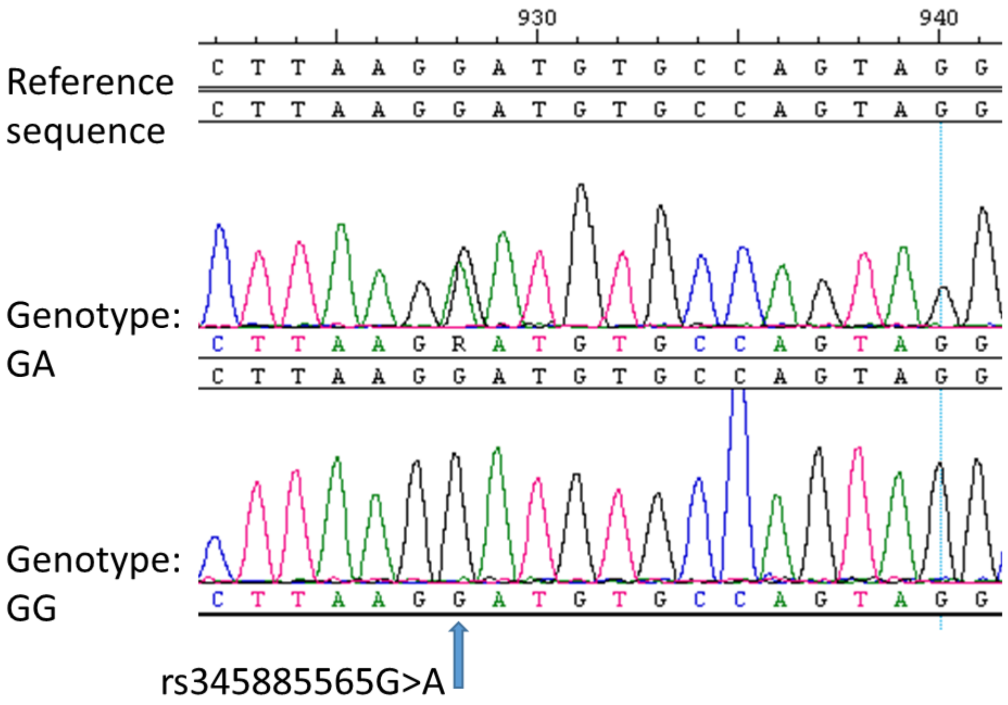

Figure S3: Sanger sequencing results for *TUBA4A* gene. Chromatograms showing the promoter polymorphism and two genotypes identified—GA and GG.

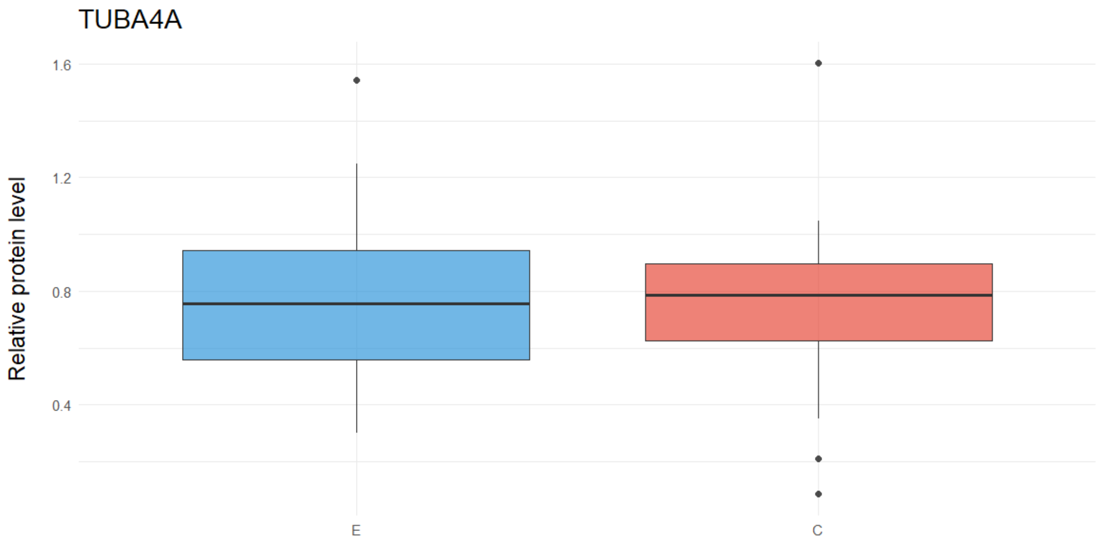

Figure S4: Relative TUBA4A protein expression level. Box plot representing the densitometric analysis of TUBA4A protein level in the experimental group (E, n = 16) and control group (C, n = 16), normalised to the GAPDH protein as a loading control. The horizontal line inside the box indicates the median, the whiskers represent the non-outlier range, and black dots indicate statistical outliers.
